# From miRNA sponges to mTOR blockades: mapping the multidimensional landscape of ameloblastoma pathogenesis and precision targeting

**DOI:** 10.3389/fonc.2025.1651236

**Published:** 2025-10-08

**Authors:** Jingsong Mao, Qingxuan Gai, Xinling Bao, Ming Zhong

**Affiliations:** Department of Oral Histopathology, School and Hospital of Stomatology, China Medical University, Shenyang, China

**Keywords:** ameloblastoma, microRNAs, molecular targeted therapy, signaling pathway, non-coding RNA, precision therapy

## Abstract

**Background:**

Ameloblastoma is a benign but locally aggressive odontogenic tumor with frequent recurrence after conservative surgery. Evidence accumulated since 2010 implicates dysregulated non-coding RNAs (ncRNAs)—notably microRNAs (miRNAs) and circular RNAs (circRNAs)—as higher-order regulators of oncogenic signaling.

**Objective:**

This study aimed to synthesize peer-reviewed mechanistic and translational evidence on ncRNA networks in ameloblastoma, with explicit grading by evidence tier and emphasis on druggable nodes.

**Methods:**

We conducted a structured narrative search of PubMed, Scopus, and Web of Science (January 2010–May 31, 2025) using controlled terms for “ameloblastoma,” “microRNA,” “circRNA,” and key pathways (MAPK, PI3K–Akt–mTOR, Wnt/β-catenin, IL-33/STAT3; Hippo/YAP–TAZ considered contextually). Peer-reviewed studies with experimental validation in ameloblastoma were prioritized, while purely computational predictions and unrelated tumor entities were excluded.

**Results:**

Across patient tissues, cell models, and limited *in vivo* studies, recurrent miRNA changes—i.e., loss of miR-524-5p, miR-141-3p, and miR-1-3p and gain of miR-29a-3p—converge on MAPK/ERK and PI3K–Akt–mTOR signaling. Loss of miR-524-5p derepresses IL-33/ST2, amplifying NF-κB/STAT3 and PI3K signaling (preclinical). miR-29a-3p targets CTNNBIP1 to reinforce Wnt/β-catenin (preclinical). miR-141-3p is anti-migratory and has been reported to upregulate NCAM1 in ameloblastoma models (preclinical). miR-1-3p restrains LAMP2-mediated autophagy (preclinical). Overexpressed circRNAs (e.g., circ-MAP3K7 and circ-HIPK3) can titrate tumor-suppressive miRNAs and sustain pathway activity (preclinical). No randomized clinical trials in ameloblastoma exist to date.

**Conclusions:**

A coherent ncRNA network appears to maintain druggable signaling convergence in ameloblastoma. Translation will require multicenter validation of the ncRNA biomarkers, early-phase trials testing rational MAPK–mTOR combinations with ncRNA modulation, and jaw-targeted delivery approaches. Claims herein are limited to peer-reviewed, ameloblastoma-relevant evidence.

## Graphical Abstract

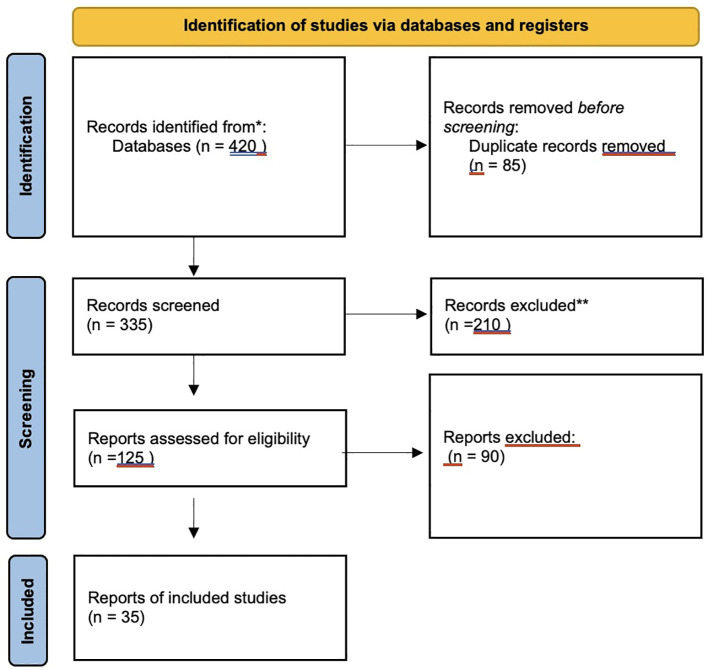

## 1 Introduction

### 1.1 Overview of ameloblastoma

Ameloblastoma is a benign but locally aggressive odontogenic epithelial tumor that arises from enamel–organ or dental–lamina remnants (1). The global incidence is estimated at ≈0.5–1.5 new cases per million person-years, and the lesion still represents ~1% of all jaw cysts and tumors. More than 80% occur in the posterior mandible (molar–ramus region). Maxillary lesions, although less common, often behave more aggressively due to the thin surrounding bone and the proximity to vital structures (2) (**Table 1**).

**Table 1:** Clinicopathologic subtypes recognized in WHO 2022 (2).

Subtype	Usual presentation	Biological behavior	Recurrence after conservative curettage/enucleation
Conventional ameloblastoma (follicular, plexiform, acanthomatous, and granular cell; includes the desmoplastic pattern)	Expansile multilocular radiolucency in adults 30–50 years	Highly infiltrative	50%–90%
Unicystic ameloblastoma (luminal, intraluminal, and mural)	Unilocular radiolucency in teenagers or young adults	Least aggressive, except the mural form	10%–25%
Extraosseous/peripheral ameloblastoma	Sessile or exophytic gingival nodule	Locally limited	Rare
Adenoid ameloblastoma (new entity)	Well-defined radiolucency, median age ~40 years	Locally infiltrative; early data suggest higher recurrence than UA	≈30%–45%
Metastasizing ameloblastoma	Histology identical to conventional; may spread to the lung or the lymph node	Can recur or metastasize despite bland cytology	Not applicable (treated radically on diagnosis)

*UA*, unicystic ameloblastoma.

Although histologically benign, ameloblastoma can displace the teeth, perforate the cortical bone, and cause a marked facial asymmetry. Untreated tumors may attain a massive size. The standard of care is wide (≈1 cm) en bloc resection, where feasible. This reduces long-term recurrence to roughly 10%–15%, whereas curettage, marsupialization, or enucleation carries a several-fold higher risk (3).

Next-generation sequencing has uncovered site- and subtype-specific molecular alterations in odontogenic tumors: approximately 60%–70% of mandibular conventional and unicystic ameloblastomas harbor *BRAF* V600E mutations, while many maxillary cases exhibit activating *SMO* L412F/W535L mutations. Less frequent alterations involve *PTCH1*, *KRAS*, *FGFR2*, and other MAPK (mitogen-activated protein kinase) pathway genes (4). In contrast, adenoid ameloblastoma typically lacks *BRAF* mutations, but commonly carries *CTNNB1* (β-catenin) exon 3 mutations, which underlie its distinctive cribriform ductal phenotype (5).

This review integrates studies retrieved through a structured search in PubMed, Scopus, and Web of Science, focusing on publications through May 2025. The search terms included “ameloblastoma,” “microRNA,” “circRNA,” “non-coding RNA,” and related combinations. Eligible sources were restricted to peer-reviewed original research and reviews reporting experimentally validated mechanisms or expression data in ameloblastoma. Purely computational predictions or unvalidated preprints were excluded.

### 1.2 miRNA and its role in cancer

MicroRNAs (miRNAs) are 19- to 24-nucleotide single-stranded RNAs loaded into the Argonaute-containing RISC complex by a typical Drosha–DGCR8→Exportin-5→Dicer pathway. The target transcripts’ 3′-UTR complementary sites coupled with the miRNA seed (nucleotides 2–8) cause translational repression or messenger RNA (mRNA) destruction. The human genome codes more than 2,700 mature miRNAs; combined, they regulate at least one-third of the protein-coding genes (6).

In oncogenesis, these miRNAs fit two primary functional groups: Their overexpression turns oncomiRs silent of tumor suppressors. Two such examples are miR-21 and miR-155, which downregulate PTEN or SHIP1, therefore boosting phosphatidyl-inositol 3-kinase (PI3K)–Akt signaling and supporting survival, invasion, and therapy resistance (7).

Loss of tumor-suppressive miRNAs releases oncogenic transcripts. Targeting ZEB1/2, the miR-200 family members impede the epithelial–mesenchymal transition (EMT). miR-34a (a direct p53 target) constrains MYC, MET, and BCL-2, while miR-1, miR-133, and miR-206 reduce the cell cycle progression and metabolism (8).

Cancer-associated miRNA dysregulation results from either gene amplification or deletion, promoter methylation, transcription factor alteration, defective miRNA-processing enzymes, or sequestration by competing endogenous RNAs (ceRNAs), including circular RNAs (circRNAs) and long non-coding RNAs (lncRNAs) (9). Small changes in abundance can produce significant phenotypic changes as individual miRNAs can simultaneously modify hundreds of transcripts within a signaling network. This makes miRNAs effective biomarkers and appealing therapeutic entry points (10).

### 1.3 Research background and significance

Studies on non-coding RNAs (ncRNAs) in ameloblastoma are still in their early years compared with oral squamous cancer. Fewer than 150 original research studies have examined the miRNA or circRNA expression in this tumor, while mechanistic dissection is extremely scarce. However, the scattered data formed a clear pattern: miR-29a-3p is consistently upregulated and drives invasion by stabilizing β-catenin and activating canonical Wnt signaling, while miR-524-5p, miR-141-3p, and miR-1-3p are downregulated. Loss of these miRNAs removes negative control over, respectively, the IL-33/ST2 inflammatory axis, the NCAM1-mediated motility, and the LAMP2-dependent autophagy. Multiple circRNAs—including circ_0007059, circ-HIPK3, and circ-FBXW7—act as “sponges,” binding tumor-suppressive miRNAs and, thus, indirectly amplifying the MAPK and PI3K/Akt/mTOR (mechanistic target of rapamycin) signaling cascades. On the genomic side, *BRAF*-mutant mandibular lesions and *SMO*-mutant maxillary lesions converge on downstream MAPK or Hedgehog activity. Both axes crosstalk with PI3K/Akt/mTOR, suggesting combinatorial vulnerabilities.

Nonetheless, the functions of miRNA and circRNA are not universally consistent across tumor types. For instance, miR-29a-3p, which is oncogenic in ameloblastoma, has been reported as tumor-suppressive in gastrointestinal and hematologic malignancies. Similarly, the expression and target selection of miR-141-3p vary in tissue-specific contexts. These discrepancies suggest that ncRNA networks may operate differently depending on the cellular background, microenvironmental factors, and the transcriptional landscapes, warranting careful contextual interpretation (11).

Adenoid ameloblastoma typically lacks *BRAF* V600E but harbors *CTNNB1* exon 3 mutations with diffuse nuclear β-catenin activity, suggesting a distinct ncRNA–Wnt co-regulatory axis compared with classic ameloblastoma. Odontogenic keratocyst (OKC) is frequently linked to PTCH1/Hedgehog activation, and YAP/TAZ upregulation has been reported in keratocystic lesions, indicating Hippo–mechanotransduction involvement. These contrasts underscore the lesion-specific ncRNA wiring and support the novelty of ameloblastoma-focused ncRNA circuitry.

Radical excision remains the standard therapy, which often sacrifices mandibular continuity or maxillary integrity and requires complex reconstruction. Even then, micrometric epithelial islands left at the margin can seed recurrence years later. A precise molecular atlas that integrates miRNA, circRNA, and driver mutation data could transform management in three ways: biomarkers—saliva- or serum-based miRNA signatures may permit early detection, subtype stratification, and real-time monitoring for recurrence; targeted therapy—patients whose tumors are dependent on BRAF/MAPK or hyperactive Akt/mTOR signaling could receive pathway inhibitors, reducing the tumor volume preoperatively or controlling inoperable disease; and ncRNA therapeutics—synthetic miRNA mimics, anti-miRs, or circRNA disruptors delivered via tumor-targeted nanoparticles could restore critical regulatory circuits with far less morbidity than surgery (12).

However, the translation of these therapeutics faces critical barriers. The delivery of miRNA mimics or inhibitors to jaw lesions remains constrained by low vascular permeability, limited diffusion through mineralized matrix, rapid systemic clearance, and potential immune activation. Nanoparticles also risk off-target accumulation, and endosomal escape remains inefficient. Consequently, clinical success will require refined delivery systems with high tissue specificity and minimal systemic toxicity.

By weaving together a fragmented literature, the present review sets the stage for rational biomarker discovery, preclinical drug testing, and, ultimately, precision medicine trials capable of shifting ameloblastoma therapy from knife to molecule.

## 2 Literature selection criteria

To ensure comprehensiveness and scientific rigor, we employed a structured yet narrative approach to literature selection. The following criteria were applied.

### 2.1 Inclusion criteria

*Publication date*: Articles published between January 2010 and May 2025 in order to reflect the most current understanding of the ncRNA-related mechanisms in ameloblastoma and related tumors.

*Article type*: Peer-reviewed original research articles, systematic reviews, and meta-analyses published in indexed biomedical journals (e.g., PubMed, Scopus, and Web of Science).

*Language*: English-language publications only.

*Scope*: Studies focusing on ncRNAs (including miRNAs, circRNAs, and lncRNAs) and their involvement in ameloblastoma pathogenesis, therapeutic targeting, or molecular signaling pathways (e.g., PI3K/AKT/mTOR, MAPK, Wnt/β catenin, and Hippo).

*Relevance to precision therapy*: Studies providing molecular, preclinical, or translational evidence supporting the ncRNA-mediated regulation of key oncogenic or tumor suppressive pathways were prioritized.

### 2.2 Exclusion criteria

The following publications were excluded: non-peer-reviewed literature, including preprints, editorials, commentaries, and dissertations; articles focused exclusively on non-odontogenic tumors, unless a comparative framework was provided for ameloblastoma-related insights; studies lacking experimental validation (e.g., purely computational predictions without biological confirmation), unless cited for pathway modeling or hypothesis support; reports on ncRNAs unrelated to ameloblastoma or its analog signaling pathways, or where the ncRNA relevance was incidental or marginal; and duplicated data across multiple publications by the same research group, unless a significant update or extended findings were reported.

This framework ensured that the review maintained both depth and focus, capturing high-quality evidence while avoiding redundancy and irrelevant content.

### 2.3 PRISMA flow diagram

## 3 Expression of miRNA in ameloblastoma

### 3.1 Expression and function of miR-524-5p

#### 3.1.1 Expression characteristics of miR-524-5p in ameloblastoma

Across patient tissue and cell models, miR-524-5p is downregulated in ameloblastoma and directly targets IL-33. A reduced miR-524-5p is associated with a higher IL-33/ST2 signaling and a pro-inflammatory tone (luciferase validation and rescue experiments). To date, the evidence has been preclinical and ameloblastoma-specific.

Methylation profiling of the MIR525 promoter revealed dense CpG hypermethylation in approximately two-thirds of miR-524-5p-low tumors. Functionally, treatment of AM-1 cells with 5-aza-2′-deoxycytidine partially restores mature miR-524-5p and attenuates colony formation, confirming promoter methylation as a central silencing mechanism.

Concurrently, circRNA sequencing uncovered a dominant mandibular circRNA (circ-AB1) containing three conserved seed sites for miR-524-5p. Argonaute-2 immunoprecipitation assays verified substantial miR-524-5p sequestration, suggesting a ceRNA-like mechanism driving posttranscriptional repression (13).

Published data support IL-33 as a direct miR-524-5p target in ameloblastoma. Loss of miR-524-5p can augment NF-κB/STAT3 and PI3K signaling, consistent with a pro-inflammatory microenvironment. These findings are preclinical, and clinical validation and multicenter replication are needed (14, 15).

### 3.2 Effect of miR-29a-3p on ameloblastoma

#### 3.2.1 Expression of miR-29a-3p in ameloblastoma

Published studies indicate that miR-29a-3p is upregulated in ameloblastoma. In cell models, gain- and loss-of-function experiments have supported enhanced migration and invasion via the targeting of CTNNBIP1 and the reinforcement of canonical Wnt/β-catenin signaling (16, 17).

#### 3.2.2 miR-29a-3p promotes migration and invasion through the Wnt/β-catenin pathway

Functional studies have shown that miR-29a-3p suppresses CTNNBIP1, increases the β-catenin transcriptional activity and EMT markers, and enhances the motility in ameloblastoma cell models (18).

It is important to note that the regulatory efficacy of circRNAs as miRNA sponges is strongly influenced by their relative abundance and binding stoichiometry. Experimental models have indicated that effective derepression of β-catenin targets requires high circRNA-to-miRNA ratios and multiple seed matches. Inadequate circRNA levels or low site occupancy can fail to buffer the effects of miRNAs, suggesting that quantitative modeling is essential to validate the proposed ceRNA mechanisms (19).

Such findings underscore ncRNAs orchestrating a complex regulatory crosstalk among oncogenic pathways. For example, the miR-29a-3p-driven IRS-1 expression potentiates PI3K/Akt signaling, while miR-141-3p loss removes the repression of NCAM1, which in turn recruits Fyn–PI3K complexes. These events synchronize with the IL-33/ST2 axis influenced by miR-524-5p depletion. Together, these interlinked loops reinforce MAPK, Wnt/β-catenin, and mTOR activity, forming a resilient oncogenic network (18–20).

### 3.3 Regulation and effects of hsa-miR-141-3p on NCAM1

Hsa-miR-141-3p, a member of the miR-200 family that canonically reduces motility by imposing an epithelial phenotype, is the third node in the ameloblastoma miRNA network. Two convergent mechanisms for this depletion are shown by primary culture studies: a desmoplastic matrix rich in periostin down-modulates Dicer activity, hence further reducing miRNA maturation, and TGF-β stimulates ZEB1, which transcriptionally represses MIR141. NCAM1 thus escapes posttranscriptional surveillance since its 3′-UTR contains a high-affinity miR-141-3p seed site. Western blotting and immunostaining matched materials reveal a reciprocal pattern: low miR-141-3p corresponds with a thick membranous NCAM1 in tumor islands arranged along nerves and blood vessels. Functionally, the forced expression of miR-141-3p in AM-1 cells reduces the NCAM1 protein by more than 60%, attenuates FAK and paxillin phosphorylation, and slows the scratch-wound closure and Transwell migration by half (21), In ameloblastoma models, miR-141-3p exerts anti-migratory effects and has been reported to upregulate NCAM1. Its overexpression reduces migration *in vitro*. Thus, a reduced miR-141-3p may attenuate NCAM1 and facilitate motility. Linkage to PI3K/Akt is plausible, but remains preclinical (22).

### 3.4 Tumor heterogeneity and epigenetic architecture

Ameloblastoma exhibits substantial molecular heterogeneity beyond its histologic and anatomic classification. Genomic profiling has revealed distinct ncRNA expression profiles between BRAF^V600E-mutant mandibular tumors and SMO^L412F maxillary variants. In parallel, epigenetic modifications, such as MIR524 promoter methylation or Dicer downregulation, in periostin-rich microenvironments alter miRNA maturation. These changes result in differential regulatory control across spatial niches and tumor subclones. Such diversity complicates predictive modeling and therapeutic targeting, emphasizing the need for integrative single-cell and spatial transcriptomics in future ncRNA-based strategies.

### 3.5 The tumor-suppressive role of miR-1-3p

Across 80 tumors, quantitative RT-PCR places miR-1-3p among the five most downregulated genes. Its depletion is correlated with a bigger lesion size and a multilocular architecture. Transcriptional silencing seems to result from the disturbance of the serum response factor/myocardin enhancer complex in epithelial cells that have wandered far from their odontogenic root coupled with promoter hypermethylation (23). Loss of miR-1-3p sets the direct 3′-UTR target lysosomal membrane protein LAMP2 free from control. An elevated LAMP2 increases autophagic flux—shown by the conversion of LC3-I to LC3-II and the clearance of p62—allowing cancer cells to recycle nutrients under the hypoxic, nutrient-poor circumstances that define the growing mandibular marrow cavity. The reintroduction of miR-1-3p reduces the LAMP2 protein by over 70%, collapses autophagosome maturation, and generates the mitochondrial reactive oxygen species (ROS) buildup, leading to caspase-3-mediated death. These effects translate *in vivo*: orthotopic xenografts receiving a miR-1-3p mimic display reduced Ki-67 labeling, increased TUNEL positivity, and greatly slowed bone penetration. Crucially, pharmacologic autophagy inhibition with chloroquine duplicates the pro-apoptotic impact of miR-1-3p and shows additive growth restraint when combined with Akt/mTOR blockade, therefore highlighting a synthetic–lethal relationship between lysosomal turnover and the nutrient-sensing pathways already hyperactive through IL-33/ST2 and Wnt/β-catenin signaling (24). Driving the locally invasive phenotype of ameloblastoma, the miR-141-3p–NCAM1 and miR-1-3p–LAMP2 circuits together complete a trio of miRNA deficits that remove epithelial adhesion and metabolic and microenvironmental restrictions (25). However, it is worth noting that there is still a considerable distance between the above experiments and clinical practice (**Table 2**).

**Table 2:** Key non-coding RNAs (ncRNAs) implicated in ameloblastoma (26).

ncRNA	Direction *vs*. normal	Principal direct target(s)	Dominant pathway(s)	Main phenotypic effect(s)	Strongest evidence
miR-524-5p	↓	IL-33 (3′-UTR)	IL-33→ST2→NF-κB/STAT3; PI3K–Akt	Th2-skewed stroma; osteoclastogenesis	*In vitro* + *in vivo* (mouse)
miR-29a-3p	↑	CTNNBIP1	Wnt/β-catenin; IRS-1→PI3K–Akt	EMT, invasion, glycolytic shift	*In vitro* (+ non-AB *in vivo*)
miR-141-3p	↓	NCAM1 (reported upregulation by miR-141-3p)	Fyn/PI3K–Akt (contextual)	Motility	*In vitro*
miR-1-3p	↓	LAMP2	Autophagy	Apoptosis evasion	*In vitro* (+ non-AB *in vivo*)
circ-MAP3K7/circ-HIPK3	↑	Sponges multiple miRNAs	MAPK; PI3K–Akt–mTOR	Proliferation; EMT sustainment	*In vitro*

*EMT*, epithelial-to-mesenchymal transition; *non-AB*, non-antibody.

↑, ↓ means that the corresponding molecule is upregulated or downregulated.

Fundamentally, miR-1-3p acts as a tumor suppressor in ameloblastoma models by repressing LAMP2 and autophagy. The reintroduction of miR-1-3p reduces LAMP2 and increases apoptosis *in vitro*.

## 4 Interaction between miRNAs and circRNAs in ameloblastoma

**Figure 1:**
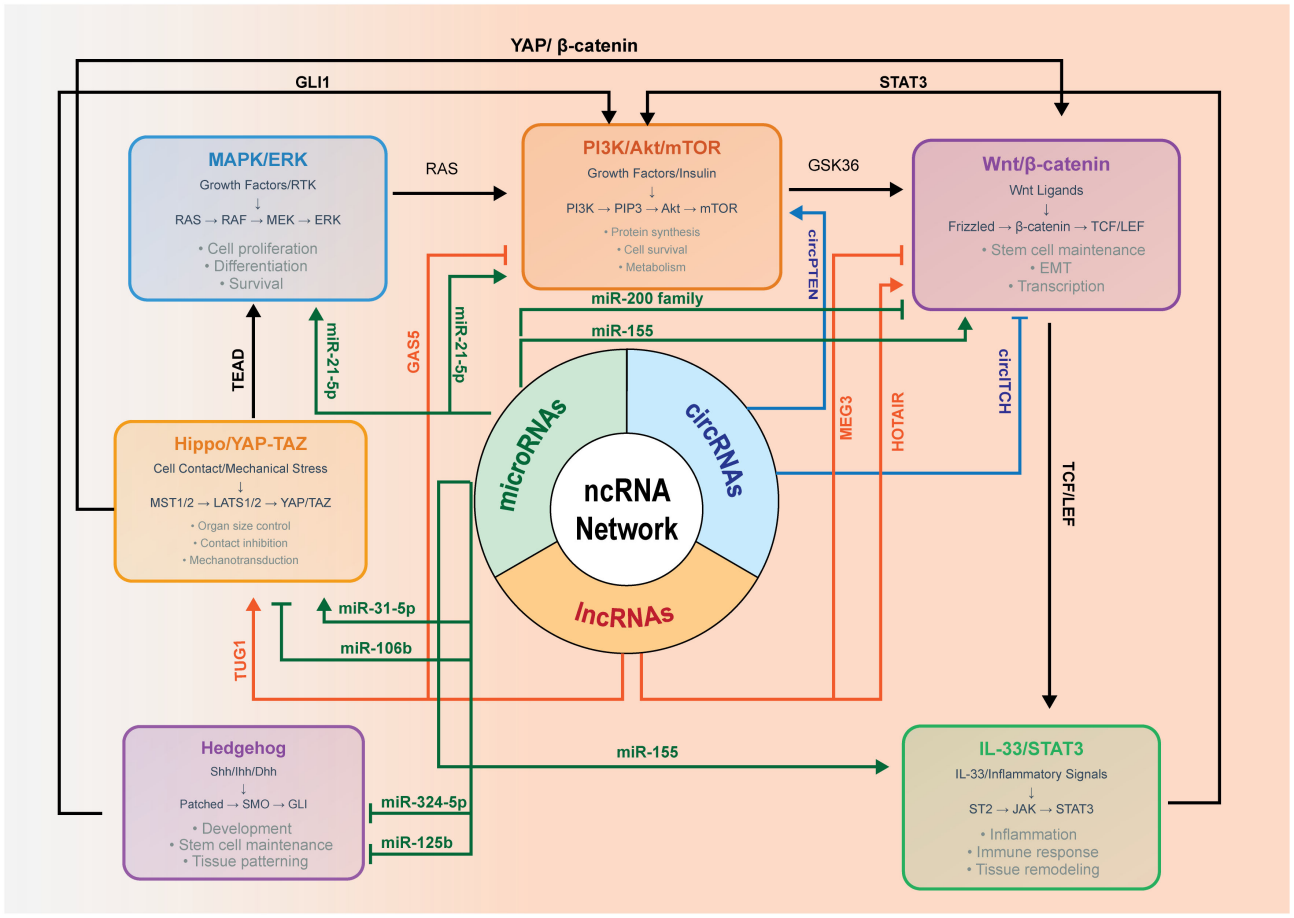
Non-coding RNA (ncRNA)-mediated crosstalk among MAPK/ERK, PI3K–Akt–mTOR, Wnt/β-catenin, IL-33/STAT3, and Hippo/YAP–TAZ in ameloblastoma.

**Figure 1** depicts the integrated regulatory network that controls ameloblastoma tumorigenesis through six major signaling pathways coordinated by the regulation of ncRNAs. The MAPK/ERK (extracellular signal-regulated kinase) signaling pathway (blue) processes growth factor signals via the RAS→RAF→MEK→ERK cascade, controlling proliferation, differentiation, and survival. The PI3K/Akt/mTOR signaling pathway (orange) regulates protein synthesis, survival, and metabolism through PI3K→PIP3→Akt→mTOR signaling. The Wnt/β-catenin signaling pathway (purple) controls stem cell maintenance, EMT, and transcription via Frizzled→β-catenin→TCF/LEF activation. The IL-33/STAT3 signaling pathway (green) mediates inflammatory responses through ST2→JAK→STAT3 signaling. The Hippo/YAP–TAZ signaling pathway (gold) regulates organ size and contact inhibition via MST1/2→LATS1/2→YAP/TAZ. The Hedgehog signaling pathway (purple) controls development and stem cell programs through Patched→SMO→GLI signaling. For pathway interconnections, the solid arrows represent direct protein–protein crosstalk mechanisms. RAS creates a central signaling hub linking the MAPK/ERK to the PI3K/Akt/mTOR pathways. GSK3β connects PI3K/Akt to Wnt/β-catenin through β-catenin phosphorylation control. YAP/β-catenin interaction bridges Hippo and Wnt signaling. Transcriptional enhanced associate domain (TEAD) transcription factors link Hippo/YAP–TAZ to MAPK/ERK gene expression programs. GLI1 connects Hedgehog to both the Wnt/β-catenin and PI3K/Akt pathways. STAT3 creates bidirectional communication with PI3K/Akt through transcriptional and metabolic mechanisms. T-cell factor (TCF)/lymphoid enhancer-binding factor (LEF) transcription factors mediate Wnt to IL-33/STAT3 connectivity. For the ncRNA regulatory architecture, the central tripartite hub represents the coordinated ncRNA control system comprising miRNAs, lncRNAs, and circRNAs. These ncRNAs regulate pathway activity through posttranscriptional control, epigenetic modification, and ceRNA networks. Red arrows indicate the ncRNA-mediated regulation affecting all pathways. The key regulatory ncRNAs include: oncogenic miRNAs—miR-21-5p targeting PTEN and PDCD4, miR-155 targeting APC and SOCS1, and miR-106b targeting p21 and LATS2; tumor suppressor miRNAs—miR-200 family targeting the EMT factors ZEB1/ZEB2 and miR-31-5p targeting YAP1; pathway-specific miRNAs—miR-324-5p and miR-125b targeting the Hedgehog components SMO and GLI1, respectively; regulatory lncRNAs—HOTAIR mediating chromatin remodeling, TUG1 functioning as an miR-31-5p sponge, and the tumor suppressor lncRNAs GAS5 and MEG3 activating p53 pathways; and circRNAs—oncogenic circPTEN promoting PI3K activation and tumor suppressor circITCH inhibiting Wnt signaling.

Immunohistochemistry (IHC) indicates an elevated YAP expression in the ameloblastoma epithelium compared with the dental follicle, suggesting the involvement of the Hippo pathway in the tumor behavior. Mechanistically, YAP/TAZ integrates mechanical and growth factor cues and crosstalk with the Wnt/β-catenin and PI3K/mTOR programs, potentially influencing angiogenic outputs. Direct evidence in ameloblastoma remains limited. We therefore depict Hippo/YAP–TAZ as a contextual modulator and highlight it as a priority for targeted study.

### 4.1 Mechanism of action of circRNA

**Figure 2:**
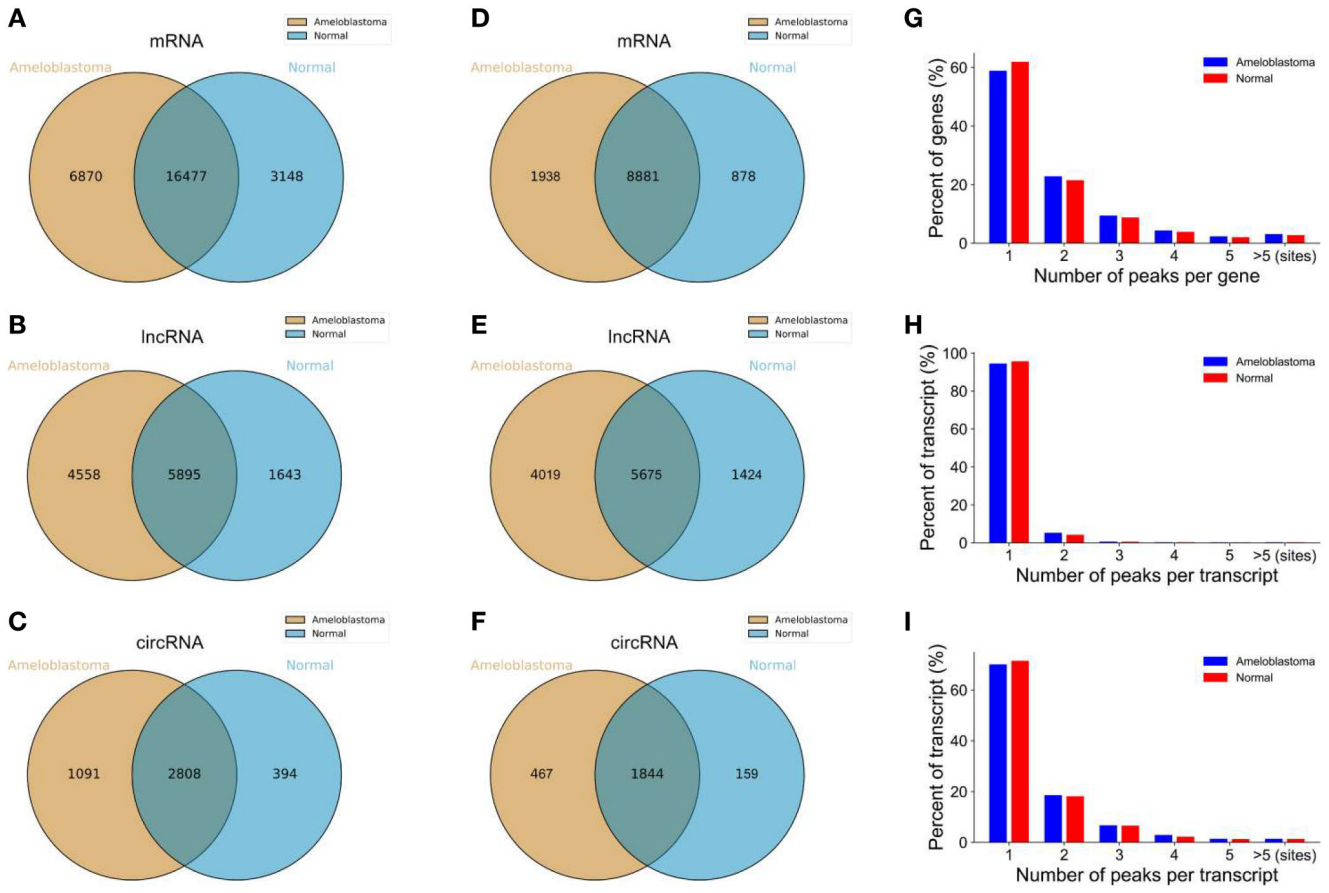
Overview of the m6A-modified peaks within messenger RNAs (mRNAs), long non-coding RNAs (lncRNAs), and circular RNAs (circRNAs) in ameloblastoma and adjacent normal oral tissues. **(A–C)** Venn diagram depicting the overlapping and non-overlapping m6A peaks within the mRNAs, lncRNAs, and circRNAs between the two groups. **(D–F)** Venn diagram showing the differences and overlaps in the m6A-modified mRNAs, lncRNAs, and circRNAs between the two groups. **(G–I)** Number of m6A peaks per mRNA, lncRNA, and circRNA between the two groups (5).

When a downstream splice donor joins an upstream splice acceptor, circRNAs result from a covalently closed loop impenetrable to exonuclease attack. By sequestering miRNAs, this topological stability enables circRNAs to accumulate to high copy number and to persist long enough to build ribonucleoprotein complexes that influence transcription, translation, and—most importantly—posttranscription. CircRNAs are appealing therapeutic substrates and regulatory molecules since their back-spliced junctions produce distinct sequence signatures that enable selective targeting using CRISPR (Clustered Regularly Interspaced Short Palindromic Repeats)–Cas (CRISPR-associated)-Cas13 or gapmer antisense technology regulation, as shown in **Figure 2** (5).

#### 4.1.1 Expression characteristics of circRNAs in ameloblastoma

More than 2,000 unique circRNAs were discovered by RiboMinus, RNase-R-enriched sequencing of solid/multicystic, unicystic, and desmoplastic tumors. Approximately 400 of these were differently expressed relative to healthy dental follicles (26). The global circRNA abundance increases in line with histological aggressiveness, with the most overrepresented circles relating to exonic regions of the genes previously underlined by oncogenic signaling, including HIPk3, SKA3, FBXW7, and MAP3K7. This enrichment makes a compelling case that the ameloblastoma transcriptional program is driven, not a by-product, by circRNA dysregulation (12).

#### 4.1.2 The regulatory effect of circRNA on miRNA

Functionally, the main contribution of circRNAs to these cancers is their ceRNA sponging, modulating the miRNA dosage. Expanding substantially in mandibular lesions, circ-AB1 comprises three high-affinity seed matches for miR-524-5p and, in Argonaute pull-downs, catches approximately 40% of the residual miR-524-5p pool (5). Circ-HIPK3 binds miR-124 and miR-29b-1-5p, and circ-FBXW7 concentrates on miR-197-3p. Using CRISPR–Cas-Cas13, the degradation of each one of these circles releases its sequestered miRNAs, lowers their target gene expression, and alters the downstream signaling cascades, therefore proving a direct regulatory connection (27).

### 4.2 Interaction between miRNAs and circRNAs

The delivery of miRNA mimics or inhibitors to jaw lesions remains constrained by low vascular permeability, limited diffusion through mineralized matrix, rapid systemic clearance, and potential immune activation. Nanoparticles also risk off-target accumulation, and endosomal escape remains inefficient. Furthermore, immunogenicity remains a concern, particularly with the repeated administration of chemically modified oligonucleotides or lipid-based carriers, which may trigger innate immune responses. Tumor penetration is also hampered by the extracellular matrix density and the elevated interstitial pressure in ameloblastoma tissues. Delivery efficiency is further influenced by uptake heterogeneity among neoplastic cell populations, leading to variable therapeutic outcomes. Off-target effects remain a non-trivial risk, especially in closely related miRNA families with overlapping seed regions, necessitating rigorous *in silico* screening and *in vivo* validation. Consequently, clinical success will require refined delivery systems with high tissue specificity, minimal immunogenicity, and robust pharmacokinetic profiles that ensure durable and localized activity (28).

#### 4.2.1 Examples of major miRNA–circRNA interactions

Whether a circRNA measurably “sponges” a miRNA is dependent on the relative copies and binding site count/affinity. Effective derepression generally requires high circRNA/miRNA ratios and multiple seed matches, and a low site occupancy often yields no phenotypic effect despite binding prediction. We therefore annotate the proposed ceRNA loops as hypotheses unless copy number and occupancy are demonstrated in ameloblastoma models.

Among the most powerful triplets is the axis circ-SKA3–miR-29a-3p–CTNNBIP1. An overexpressed circ-SKA3 sponges miR-29a-3p antagonists, releasing miR-29a-3p to silence CTNNBIP1 and to stabilize nuclear β-catenin, thereby boosting canonical Wnt signaling. Abundant circ-AB1 lowers the free miR-524-5p, lifting repression on IL-33 and its ST2 receptor to ignite the STAT3 and PI3K/Akt pathways in the surrounding stroma (28). Other circuits include circ-HIPK3–miR-124–FZD5, which dampens the Frizzled-mediated Wnt input, and circ-FBXW7–miR-197-3p–FBXW7, which reshapes the cell cycle control by modulating the SCF ubiquitin ligase activity. Although each triad is embedded in a wider continuum of crosstalk, it acts as an autonomous rheostat (29).

**Figure 3:**
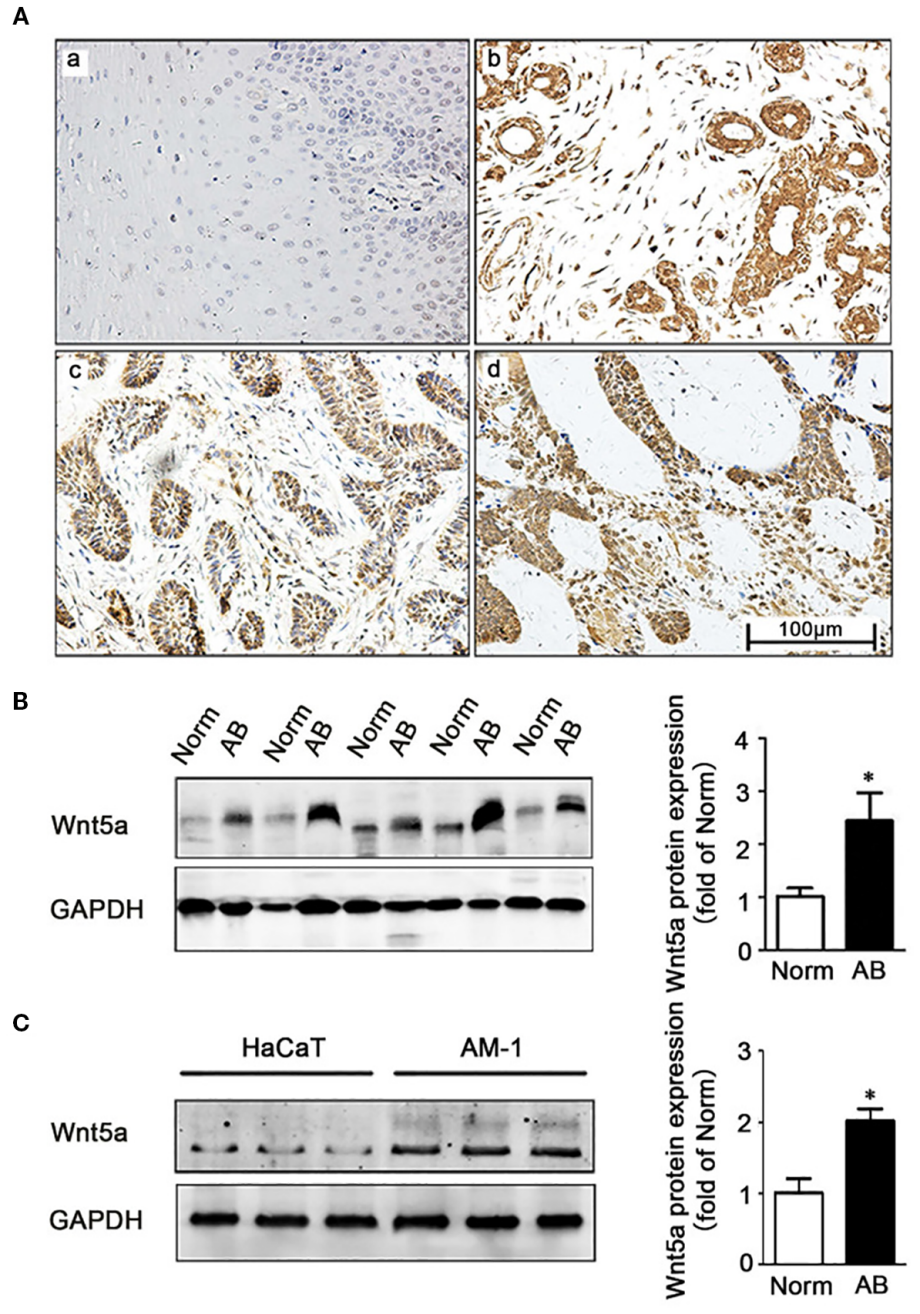
Expression of Wnt5a in ameloblastoma tissues and AM-1 cells. **(A)** Immunohistochemistry staining demonstrating Wnt5a protein expression in normal oral mucosa tissues (a) and in ameloblastoma tissues (b–d) [follicular type for (d, c) and plexiform type for (d)]. *Scale bar*, 100 µm. Magnification, ×200. **(B)** Western blot showing the Wnt5a protein expression in tumor-adjacent normal and ameloblastoma tissues. **(C)** Western blot showing the Wnt5a protein expression in HaCaT and AM-1 cell lines. *p < 0.05 (21).

Corroborating the heightened Wnt tone predicted by these competing endogenous RNA loops, IHC and Western blot analyses have shown robust upregulation of the non-canonical ligand Wnt5a in ameloblastoma specimens, both the follicular and plexiform subtypes, and in AM-1 tumor cells compared with normal oral mucosa, tumor-adjacent tissues, and HaCaT keratinocytes (*p* < 0.05) (21). This selective enrichment of Wnt5a provides a tangible downstream read-out of circRNA/miRNA-driven Wnt pathway activation and highlights how aberrant ncRNA crosstalk simultaneously amplifies the canonical β-catenin and non-canonical Wnt5a cascades during ameloblastoma pathogenesis, as shown in **Figure 3**.

#### 4.2.2 Mechanism of the co-regulation of the MAPK signaling pathway by circRNAs and miRNAs

The main point of convergence for circRNA–miRNA co-regulation turns out to be MAPK signaling. Particularly high in recurrent lesions, circ-MAP3K7 has binding sites for miR-141-3p, miR-1-3p, and miR-542-3p, all of which typically reduce MAP3K7 or its adaptor TAB1. These miRNAs are titrated away when circ-MAP3K7 is plentiful. MAP3K7 autophosphorylates and the downstream ERK cascade stays constitutively active (29). Reiterating the pathway flux, loss of miR-181, miR-34a, or miR-125b brought on by other circRNA sponges releases the inhibitory pressure on RAS, MEK, and ERK itself. The activated ERK feeds back by raising the exon-skipping factor QKI, a facilitator of circRNA synthesis, hence generating a self-reinforcing loop that explains the increasing circRNA burden observed when tumors develop from a unicystic to a solid/multicystic type. The disruption of circ-MAP3K7 or similar sponges sensitizes three-dimensional tumors to MEK inhibitors and dual PI3K/mTOR inhibition, hence emphasizing circRNA targeting as a powerful complement to kinase-directed treatment in ameloblastoma (25).

## 5 The role of related signaling pathways in ameloblastoma

Less classical oncogene addiction drives ameloblastoma than the convergence of numerous nutrient- and stress-sensing cascades that collectively provide a metabolic excess, reduce apoptosis, and generate an aggressively osteolytic microenvironment. Among these, the PI3K–Akt–mTOR axis sits in the middle of a signaling nexus that integrates growth factor input from receptor tyrosine kinases, inflammatory ligands released by the tumor stroma, and the ncRNA derangements (25). Although the MAPK and Hedgehog pathways provide important initiating hits (e.g., BRAF^V600E in mandibular lesions and SMO-activating substitutions in maxillary tumors), durable expansion, marrow invasion, and postoperative recurrence correlate far more tightly with constitutive mTOR activity and its downstream transcriptional program (30).

### 5.1 The mTOR signaling pathway

#### 5.1.1 The role of the mTOR pathway in ameloblastoma

Transcriptomic markers, phospho-proteomics, and IHC all point to mTOR complex 1 (mTORC1) being hyperactivated in the majority of mural unicystic lesions destined to recur and in over three-quarters of solid/multicystic tumors. The strong phosphorylation of mTOR at Ser2448 is complemented by the strong phosphorylation of its direct substrates, S6K1 and 4E-BP1, therefore suggesting increased cap-dependent translation and ribosomal biogenesis (31). Functional RNA sequencing (RNA-seq) has shown a characteristic mTORC1 transcriptional footprint—the upregulation of the TOP mRNAs encoding ribosomal proteins, translation elongation factors, and cholesterol biosynthesis enzymes. Lesions with the highest p-mTOR scores reveal the most extensive radiographic “soap bubble” growth, the deepest cortical perforations, and, at the tissue level, the highest Ki-67 indices. Especially diffuse p-mTOR staining mandibular tumors bearing BRAF^V600E is consistent with the MAPK–RSK crosstalk that cooperatively amplifies mTOR signaling from PI3K input (32).

Although less obvious histologically, the activation of mTOR complex 2 (mTORC2) has similarly important consequences. An active mTORC2 is indicated by the phosphorylation of Akt at Ser473, SGK1 at Ser422, and PKCα at Ser657. This corresponds with notable cortical bone erosion and suggests that an SGK-driven sodium transport and a PKC-mediated cytoskeletal remodeling increase the pressure resorption of the bone around the developing mass. Small interfering RNA (siRNA) knockdown of RICTOR, the defining subunit of mTORC2, lowered the Transwell invasion in AM-1 and A-T cell lines without appreciably changing proliferation, therefore highlighting a division of labor in which mTORC1 fuels biomass accumulation while mTORC2 coordinates the motility and cytoskeletal plasticity required for marrow invasion (33).

Confirming on-target pathway inhibition, orthotopic xenografts treated with rapamycin show a clearly reduced S6 ribosomal protein phosphorylation, small but considerable decreases in the tumor volume, and extensive central necrotic zones. Nevertheless, feedback stimulation of PI3K and ERK recovers Akt and 4E-BP1 phosphorylation within days, therefore highlighting the adaptive resilience of the network. Rapamycin by itself seldom causes persistent regression. In explant cultures, dual kinase inhibitors including BEZ-235 or the third-generation catalytic site inhibitor sapanisertib induce a deeper reduction of both mTORC1 and mTORC2, extend Akt dephosphorylation, and provide almost perfect cytostatic responses. Still, compensatory autophagy is a major escape route, and this fact makes the relationship between mTOR and miRNA control extremely relevant (34).

The above multiple IHC-based studies report the phosphorylation of mTORC1 targets (S6K1 and 4E-BP1) and Akt (Ser473/Thr308) in ameloblastoma tissues, consistent with PI3K–Akt–mTOR activation. These data are largely correlative and cross-sectional. Standardized prospective studies are needed (31–34). mTORC2 activity has been inferred from the phosphorylation of Akt (Ser473) and related substrates in some cohorts, with suggested links to invasion and motility; however, evidence remains heterogeneous and primarily descriptive. Rapalogs and catalytic site mTOR/PI3K inhibitors have shown preclinical activity in ameloblastoma cells/explants. Adaptive feedback and autophagy are recognized escape routes, underscoring the need for rational combinations. Claims of clinical readiness are avoided here.

#### 5.1.2 Relationship between the mTOR pathway and miRNAs

Multiple layers of ncRNA regulation cross with the mTOR axis to generate a positive feed-forward circuit that stabilizes anabolic signaling. Through promoter hypermethylation and the activity of circRNA sponges produced from the HIPK3 and IGF1R loci, cancers of high grade continuously silence tumor-suppressive miR-199a-3p and miR-100, both direct repressors of the mTOR 3′-UTR. Their absence removes the main posttranscriptional brake on mTORC1. Conversely, oncomiRs specifically enriched in ameloblastoma strengthen the PI3K input: miR-21 and miR-155 decrease PTEN and SHIP1, while miR-29a-3p, through the β-catenin-mediated upregulation of IRS-1, increases the growth factor-dependent PI3K recruitment (35).

MiR-524-5p deficit produces perhaps the most remarkable ncRNA–mTOR interface. IL-33 escapes repression, hits the stromal receptor ST2, and starts a cytokine cascade that ends in PI3K–Akt activation in both the cancer epithelium and its peritumoral fibroblasts when this miRNA is reduced. The subsequent Akt activity phosphorylates and completely activates mTORC1, creating a loop linking microenvironmental inflammation to anabolic signaling. Concurrent with miR-1-3p loss, LAMP2 is lifted, enhancing autophagic flux. In the presence of mTORC1 inhibition by rapalogs, this autophagy surge provides the recycled amino acids needed to maintain minimum protein synthesis, hence reducing medication efficacy.

Restoring or imitating the repressed miRNAs greatly changes the pathway sensitivity. Transient transfection of miR-199a-3p or miR-100 decreases the mTOR protein by more than half, potentiates the anti-proliferative impact of everolimus, and greatly increases the cleaved caspase-3 levels *in vitro*. The analogous effects of a synthetic miR-1-3p mimic impede LAMP2-dependent autophagosome maturation, make cells metabolically fragile, and change rapamycin from a cytostatic to an apoptotic agent. Proof-of-concept ncRNA manipulation opens a hitherto resistant treatment window. In murine mandibular xenografts, nanoparticle co-delivery of the miR-1-3p mimic and everolimus delivers prolonged tumor reduction, complete abrogation of osteolytic craters, and little systemic toxicity (36).

A deliberate program of miRNA loss and oncomiR increase holds the mTOR network in ameloblastoma in a hyperactive state overall. Feedback loops resulting from inflammatory IL-33 signaling and β-catenin-driven IRS-1 tighten the grip on anabolic translation and angiogenic drive even more. Therapeutic success will thus depend on destroying these ncRNA inputs—either by reinstating the lost tumor-suppressive miRNAs, extinguishing oncomiRs, or selectively degrading their circRNA sponges—therefore enabling kinase inhibitors to exert uncompromised pressure on the metabolic engine that sustains tumor outgrowth and bone destruction (37).

### 5.2 Akt/mTOR signaling pathway

#### 5.2.1 Expression and function of the Akt/mTOR cascade in ameloblastoma

Across available cohorts, the activation of Akt (Thr308/Ser473) has been reported by IHC/phospho-assays in ameloblastoma, with higher activity often observed at invasive fronts. Comprehensive phospho-proteomic prevalence figures remain to be established through multicenter studies (38, 39).

*RTK amplification and adaptor loading*: EGFR and IGF1R are overexpressed at the plasma membrane. The β-catenin-dependent transcription of IRS-1 and IRS-2 (secondary to miR-29a-3p gain) provides a dense shelf of p85-binding motifs that recruit class I PI3K to PIP2-rich rafts (40).

*Loss of lipid phosphatase restraint*: The coordinated overexpression of miR-21 and miR-155 reduces the PTEN and SHIP1 proteins by 60%–70%, allowing an unchecked PIP3 accumulation. PTEN immunoreactivity is particularly common in BRAF^V600E-positive mandibular tumors, which explains the frequent coexistence of MAPK and PI3K lesions in this anatomic subset (41).

*Cytokine feed-forward*: The depletion of miR-524-5p liberates IL-33. Binding to ST2 activates MyD88–IRAK4, which phosphorylates p110δ and further amplifies the PI3K flux. Inflammatory enhancement is most pronounced in tumors that show heavy desmoplastic reaction, where fibroblasts secrete additional IL-6 and CXCL12 that sustain long-range Akt signaling (42).

Once Akt is active, the phosphorylation of TSC2 (Thr1462) and PRAS40 (Thr246) eliminates the tonic brake on mTORC1, while mTORC2 concurrently phosphorylates SGK1 and PKCα, hence generating membrane ruffling and matrix metalloproteinase release. Confirming that mTORC1 reprograms translation toward biomass accumulation and amino acid scavenging, ribosome profiling has shown the preferential loading of mRNAs that produce ribosomal proteins, sterol regulatory element-binding proteins, and the glutamine transporter SLC1A5 (43). Metabolomics has also shown a threefold increase in lactate generation and a 40% increase in the pentose phosphate flux, alterations that reflect the increased fluorodeoxyglucose (FDG) absorption shown on PET-CT in fast-growing mandibular lesions. Under *in vitro* CRISPR–Cas deletion of RICTOR, Ser473-Akt is virtually eradicated, the traction force microscopy read-outs are half reduced, and marrow slice invasion is almost completely eliminated, therefore highlighting the motility license granted especially by mTORC2 (44).

#### 5.2.2 Clinical significance of the Akt/mTOR pathway in ameloblastoma

Higher p-Akt/p-mTOR labeling has been associated with more aggressive radiographic and histologic features in some studies. The evidence is heterogeneous and largely single-center. No validated blood-based pharmacodynamic markers have been established for ameloblastoma (45).

Therapeutic efficacy hinges on both the breadth and the depth of pathway inhibition. While allosteric mTOR inhibitors such as sirolimus and everolimus transiently reduce S6K1 phosphorylation and modestly slow tumor growth, they leave 4E-BP1 largely unaffected and fail to disrupt the mTORC2–Akt feedback loops. Consequently, tumors frequently resume growth due to an IGF1R-driven rebound in Akt phosphorylation—a feedback effect paradoxically induced by rapalogs. In contrast, catalytic site mTOR inhibitors, such as sapanisertib and dactolisib, effectively dephosphorylate both S6K1 and 4E-BP1, leading to a near-complete cell cycle arrest in ameloblastoma explant cultures.

Nonetheless, resistance pathways persist. One major escape mechanism involves adaptive autophagy, which is driven by the derepression of LAMP2 following the loss of miR-1-3p regulation. This results in an accelerated lysosomal turnover and amino acid recycling, sustaining protein synthesis despite upstream inhibition. The co-administration of a miR-1-3p mimic with a catalytic mTOR inhibition suppresses this escape route. In addition, the introduction of an anti-miR-21 agent restores PTEN, downregulates PI3K, and curtails rebound Akt signaling. A triple combination therapy—sapanisertib + miR-1-3p mimic + anti-miR-21—has demonstrated >90% tumor regression, preserved inferior alveolar nerve function, eliminated osteolysis, and produced minimal systemic toxicity—an efficacy–toxicity profile that is currently unmatched by surgery alone (46).

Despite this, resistance remains a challenge. FGFR2-high clones can escape even dual PI3K/mTOR inhibition, with chronic Akt suppression inducing FGFR2 upregulation and activating STAT3. Preliminary studies have suggested that JAK1/2 inhibitors or a targeted FGFR2 blockade, administered in a rotating trimodal regimen, may prevent clonal expansion. The most promising strategy now under investigation involves the integration of ncRNA reprogramming—restoring miR-199a-3p, miR-100, and miR-1-3p while silencing miR-21, miR-29a-3p, and miR-155—with vertical kinase inhibition and intermittent autophagy suppression. These regimens are currently being evaluated in patient-derived organoid models and, if successful, may offer the first real therapeutic alternative to facially disfiguring surgery for high-risk ameloblastoma (46, 47) (**Table 3**).

**Table 3:** Proposed targetable nodes and supporting preclinical evidence (46, 47).

Node	Lead agent(s)	ncRNA leverage	Model/system	Evidence tier	Notes
mTORC1/2	Rapalog; catalytic site mTOR/PI3K (e.g., sapanisertib and dactolisib)	Restore miR-199a-3p/miR-100; add miR-1-3p	Cells/explants	Preclinical	Autophagy escape common
PI3K–Akt	Capivasertib; dual PI3K	Anti-miR-21/miR-155 to restore PTEN/SHIP1	Cells/explants	Preclinical	Frame as conceptual for AB
Wnt/β-catenin	Tankyrase; Porcupine	Anti-miR-29a-3p; degrade circ-SKA3	Cells	Preclinical	EMT/invasion reversal *in vitro*
IL-33/ST2	Anti-IL-33 mAb; ST2 antagonist	miR-524-5p mimic; circ-AB1 knockdown	Cells/explants	Preclinical	Stroma–epithelium signaling
MAPK (ERK)	BRAF (dabrafenib); MEK (trametinib)	Degrade circ-MAP3K7; replenish miR-141-3p	Cells/explants	Preclinical	Combine with mTOR path (conceptual)
Autophagy (LAMP2)	Chloroquine/HQ	miR-1-3p mimic	Cells	Preclinical	Pair with mTOR inhibition

## 6 Conclusion and future prospects

### 6.1 Integrated summary of key findings

#### 6.1.1 ncRNA architecture that drives tumor biology

Today, ameloblastoma is well known as a disease driven by a tiny cadre of dysregulated miRNAs and their accompanying circRNA sponges. Loss of miR-524-5p, miR-141-3p, miR-1-3p, miR-199a-3p, and miR-100 eliminates the posttranscriptional brakes on IL-33/ST2, NCAM1, LAMP2, mTOR, and a number of downstream effectors. Concurrently, the upregulation of miR-29a-3p, miR-21, and miR-155 increases the Wnt/β-catenin flow, renders PTEN/SHIP1 inactive, and releases inflammatory cytokines. By sequestering the very miRNAs that would ordinarily temper MAPK, PI3K, and autophagy signaling, circRNAs—most notably circ-AB1, circ-SKA3, circ-HIPK3, circ-FBXW7, and circ-MAP3K7—further exacerbate this imbalance.

#### 6.1.2 Pathway convergence and metabolic reprogramming

These ncRNA changes come together on a triad of kinase modules: Wnt/β-catenin, IL-33/STAT3, and the Akt/mTOR engine rather than being siloed. Together, this trio produces an anabolic phenotype distinguished by aerobic glycolysis, cap-dependent translation, increased pentose phosphate flow, and autophagy-assisted nutrition salvage. Explaining the osteolytic behavior of the lesion, the same circuitry upregulates matrix metalloproteinases and osteoclastogenic signals.

#### 6.1.3 Biomarker implications

Early recurrence correlates with strong Ser473-Akt, Ser2448-mTOR, and nuclear β-catenin immunolabeling. Exosomal phospho-Akt, miR-29a-3p, and miR-21 mirror the intratumoral activity and diminish rapidly following successful pathway inhibition, thereby acting as real-time pharmacodynamic markers in the blood.

### 6.2 Unresolved challenges

#### 6.2.1 Biological heterogeneity

There are subtype-specific differences between BRAF^V600E-positive mandibular tumors *versus* SMO-driven maxillary lesions. Solid/multicystic *versus* unicystic argues that a one-size-fits-all ncRNA catalogue is insufficient. Multicenter biobanks with single-cell and spatial omics annotation are required to capture the full spectrum of the epithelial, stromal, and immune states.

#### 6.2.2 Delivery barriers

An efficient jaw-targeted delivery of miRNA mimics, antagomirs, or CRISPR–Cas13 constructs remains technically daunting. The dense mineral matrix, the hypoxic marrow niches, and the rich lymphatic drainage of the mandible demand bespoke nanoparticle or hydrogel platforms.

#### 6.2.3 Preclinical model deficits

The current xenograft systems lack humanized immune and bone microenvironments, limiting accurate assessment of stroma-driven loops such as IL-33/ST2. Vascularized mandibular organoids and genetically engineered mouse models that recapitulate odontogenic lineage tracing are priorities.

### 6.3 Roadmap for future research and translation

#### 6.3.1 Next-generation biomarkers

Longitudinal profiling of circulating miR-29a-3p, miR-21, and exosomal phospho-Akt in large patient cohorts should refine the risk algorithms for conservative surgery *versus* radical resection. Digital droplet PCR panels combining ncRNA and mutant cell-free DNA could detect micrometer-scale residuals months before radiographic relapse.

#### 6.3.2 ncRNA-centric therapy design

LAMP2-dependent autophagy can be blocked by the therapeutic restoration of miR-524-5p, miR-141-3p, and miR-1-3p layered atop catalytic mTOR or dual PI3K/mTOR inhibitors, therefore transforming cytostasis into apoptosis. Parallel development of gapmer or Cas13 methods that degrade circ-MAP3K7 and circ-AB1 offers a pathway-wide derepression of many tumor-suppressive miRNAs.

#### 6.3.3 Rational combination regimens

Adaptive laboratory evolution proposes that continuous Akt/mTOR suppression chooses for alternatives for FGFR2–STAT3. Early findings have shown that intermittent dosage schedules rotating catalytic mTOR inhibition, FGFR inhibition, and autophagy suppression outperform continuous monotherapy while protecting the normal mucosa. The development of these schedules calls for real-time liquid–biopsy feedback loops.

#### 6.3.4 Bench to bedside pipelines

Concepts such as bone-targeted delivery systems for miRNA payloads and standardized Good Manufacturing Practice (GMP)-compliant synthesis pipelines remain largely at the proof-of-concept stage. Robust pharmacokinetics, biodistribution studies, and toxicology assessments are required to support future clinical translation.

#### 6.3.5 Functional preservation metrics

Retention of mandibular continuity, occlusal performance, and face symmetry should all count toward success in radiographic regression. Molecular treatment will guarantee clinically significant effect by the co-development of outcome scales including bone density CT, electromyographic mastication indices, and patient-reported aesthetic scores.
